# The Psychological Impacts of COVID-19 Pandemic among Emerging Adults: An Observational Cross-Sectional Study

**DOI:** 10.3390/ijerph19031445

**Published:** 2022-01-27

**Authors:** Fatemah Alghamdi, Amal Ashour, Lola Adeyemi, Paula Bamidele, Blessing Nwambo-Logan, Maha Alsharif, Amal M. Sindi, Nada Binmadi

**Affiliations:** 1 Psychology Department, Faculty of Art & Humanities, King Abdulaziz University, Jeddah 21589, Saudi Arabia; fatemah.alghamdi.kau@gmail.com; 2 Oral & Maxillofacial Surgery and Diagnostic Sciences Department, Faculty of Dentistry, Taif University, Taif 21974, Saudi Arabia; a.a.ashour@tudent.edu.sa; 3 Magna Carta Health, Lagos 101232, Nigeria; ladeyemi@magnacartahealth.com (L.A.); seyi@mentoringher.com (P.B.); drnwambo@magnacartahealth.com (B.N.-L.); 4 Oral Diagnostic Sciences Department, Faculty of Dentistry, King Abdulaziz University, Jeddah 21589, Saudi Arabia; mtyalsharif@kau.edu.sa (M.A.); amsindi@kau.edu.sa (A.M.S.)

**Keywords:** anxiety, coronavirus, depression, emerging adult, psychological impact, student

## Abstract

(1) Background: COVID-19 has had psychological impacts, particularly anxiety, depression, anger, and suicidal ideation, on the world’s populace, including young persons who were prone to mental health disorders even before the pandemic. We described the psychological impacts of COVID-19 among emerging adults aged 18–30 years in Saudi Arabia. (2) Methods: A cross-sectional survey was done among a randomly sampled population in Saudi Arabia between July 2020 and May 2021 using the DSM-5 Self-rated Level 1 Cross-Cutting Symptom Measure and Ask Suicide Questionnaire. Logistic regression was used to assess participant characteristics associated with reporting symptoms of mental disorders under each of the 13 domains that would warrant further investigation. (3) Results: Approximately, 91% of participants experienced different mental health symptoms; these participants are more likely to be young, female students, those who had a history of being diagnosed with a mental disorder, and those diagnosed or treated for COVID-19. (4) Conclusion: This study strongly showed an increased prevalence of mental health symptoms among young persons during the COVID-19 pandemic. Addressing the mental health burden among young persons in time using simple, self-administered screening tools linked to interventions will prevent dire consequences in the future.

## 1. Introduction

Coronavirus disease 2019 (COVID-19) has impacted the world in an unprecedented way, including Saudi Arabia. Saudi Arabia reported 523,397 confirmed cases of COVID-19, with 8212 deaths according to World Health Organization (WHO) up to 30 July 2021 [1]. COVID-19 had also put people’s minds worldwide in a state of fear and apprehension [2]. Saudi Arabia initiated system-level suppressive measures, such as closing down schools and workplaces, to limit human-to-human transmission. These measures are only successful in the short term and may need to be reinstated regularly. However, long-term suppressive measures have harmful effects on society and the economy [3].

Quarantine has negative psychological consequences, such as an increase in the prevalence of symptoms of mental health disorders and diagnosed mental health disorders. The risk of mental health disorders is higher where quarantine periods are longer; information is limited, and access to supplies is unreliable; and in the presence of boredom, frustration, and potential or actual loss of income [4]. Likewise, following the SARS outbreak in 2003, research showed an increase in stress levels and adverse mental health impacts on different populations [5].

Earlier in the pandemic, COVID-19 had similar psychological impacts, particularly an increase in the proportion of persons reporting moderate to severe psychological impact among Saudi Arabia’s populace as assessed using the Impact of Event Scale-Revised (IES-R) and Depression, Anxiety, and Stress Scale (DASS-21) [6]. Emerging adults are particularly at risk of mental health disorders as has been shown in prior studies done in 15 countries preceding the COVID-19 pandemic [7], and the risk is exacerbated during the pandemic due to the disruption of their day-to-day lives as reported in many studies [8,9]. Additionally, students have reported challenges with the shift from in-person classes to remote learning [10]. Moreover, students in Nepal, Sri Lanka, and India reported different perceptions to the possibility of acquiring transferable skills from e-learning [11].

Saudi Arabia has a population of 33 million with almost an equal gender distribution. In Saudi Arabia, close to 75% of the population is less than 35 years of age [12]. In 2016, Saudi Arabia had a university capacity of 1.7 million [13]. The DSM-5 Self-rated Level 1 Cross-Cutting Symptom Measure is useful for screening persons at high risk of mental disorders (like college/university studies) across many domains of mental illness to develop tailored interventions for such populations [14]. This study described the psychological impacts of COVID-19, including depressive and anxiety symptoms and suicidal ideation attributable to the COVID-19 pandemic, among emerging adults aged 18–30 years in Saudi Arabia using the DSM-5 Self-rated Level 1 Cross-Cutting Symptom Measure. Our study expands on the mental health disorders assessed by previous research using the Impact of Event Scale-Revised (IES-R) and Depression, Anxiety, and Stress Scale (DASS-21) [6] by focusing on an age group (18–30 years) that had predominantly higher scores on the tested scales and using a pre-clinical tool that would incorporate populations that would not ordinarily seek mental health care. We employed the Young’s theoretical framework that describes the various stages a community undergoes during a disaster (such as the COVID-19 pandemic) and their responses, which may include mental illness, substance abuse, death wishes, or suicidal ideation [15]. We hypothesized that there is more than 50% prevalence of symptoms of mental illness as rated by the DSM-5 among emerging adults aged 18 to 30 years, which would warrant further investigation during the COVID-19 pandemic.

## 2. Materials and Methods

### 2.1. Study Design

This study was an observational, cross-sectional study done in Saudi Arabia between July 2020 and May 2021 to explore the psychological impacts of COVID-19 on the population’s mental health status.

### 2.2. Study Population

Study participants included in our study had to be male or female adults, emerging adults aged 18 to 30 years who could read and write in English or Arabic and provide informed consent. This population included persons who were either COVID-19 positive or COVID-19 negative who were of sound mind at the time of completing the questionnaire. We exclude all participants with impaired cognition or in ill health and not able to consent.

### 2.3. Sample Size and Sampling Method

Using a test for finite populations [16], a population of approximately 7 million aged 18–30 years [17], a prevalence of mental disorders of 24% [6], 95% confidence level, and 5% margin of error, the sample size yielded was 280. A random sampling technique was used to recruit participants. Investigators of the study forwarded the online questionnaire to their contacts who met the criteria through the emails and WhatsApp. Both the former and latter were requested to pass the survey to their contacts who met the criteria of the study.

### 2.4. Ethical Considerations

The ethical research committee at King Abdulaziz University, faculty of dentistry, granted ethical approval and permission to conduct this study (Proposal number: 072-07-20). The informed consents were attached to the questionnaire and signed electronically by the participants.

### 2.5. Procedures for Data Collection

A random sampling technique was adopted for recruitment of the study participants. The link to the questionnaire, in English and Arabic, was sent via e-mail and WhatsApp to the contacts of the investigators and the links forwarded to remarkable number of participants. Participants started the online self-administered questionnaires using Google forms after completing the eligibility and consent sections.

We used a previously validated tool for testing for psychopathology among college students [14], the Adult DSM-5 Self-Rated Level 1 Cross-Cutting Symptom Measure questionnaire [18], and another validated tool, the Ask Suicide Questionnaire (ASQ) for suicidal risk [19], to develop our questionnaire (Supplementary Materials Data). All multiple items (8 of 13 items) in each domain (as assessed among college students) on the Adults DSM-5 Self-Rated Level 1 Cross-Cutting Symptom Measure questionnaire have acceptable to good internal consistency (0.63 to 0.89). Additionally, several domains are also positively correlated with validated measures of the same mental health construct. Example items for depression include “little pleasure or interest in doing things”, “feeling down, depressed or hopeless”, and “feeling more irritated, grouchy, or angrier than usual” [14]. The ASQ has strong psychometric properties in patients aged 10–21 years (sensitivity, specificity, and a negative predictive value of 97%, 898, and 99%). Example items from the ask suicide questionnaire include, “In the past few weeks, have you wished you were dead?” and “Have you ever tried to kill yourself?” [20].

Prior to launching the survey, a pilot test to the questionnaire on 20 participants was done to study the questions clarity and verify reliability in both languages. We pretested the data collection tool on a sample of participants who were not included in the final analysis and revised the data collection tool based on the results of the pretesting procedure. We restricted multiple responses from the same user by setting the form to only allow one entry per email address. The data were collected anonymously between July and October 2020. Furthermore, only specific users could enter data in the google form.

### 2.6. Statistical Analysis

The study used frequencies and proportions to summarize participant characteristics [21] and the DSM-5 criteria to evaluate the number of participants requiring further inquiry for different domains of symptoms of mental health disease [15]. There were 13 domains with 23 questions. Responses to each question in the Adult DSM-5 Self-Rated Level 1 Cross-Cutting Symptom Measure questionnaire are rated on a five-point scale from 0—none, 1—slight, 2—mild, 3—moderate, and 4—severe. For three domains (psychosis, substance abuse, and suicide ideation), a score of 1 or more or a response in the affirmative would warrant further investigation. For the other ten domains (depression, anxiety, mania, psychosomatic symptoms, dissociation, personality dysfunction, anger, sleep problems and memory problems, repetitive thoughts, and behaviors), a score > 2 on any question would warrant further investigation [15] (Table 1). Participants were described as being diagnosed with a mental disorder during the pandemic based on their response to the question, “If you were diagnosed with a mental health issue by a medical doctor or visited a hospital for a mental health issue DURING the COVID-19 pandemic, what was it? (If not write—None)”.

Participants were categorized as either having symptoms that would warrant further investigations (a score of 1 or more or a response in the affirmative on domains of psychosis, substance abuse, and suicide ideation domains or a score of 2 or more on other 10 domains) or as having no symptoms that would warrant further diagnosis (a score of 0 or more or a response in the negative on domains of psychosis, substance abuse, and suicide ideation domains or a score of less than 2 on other 10 domains, respectively). Chi-square statistics were used to compare participants who reported symptoms that warranted further investigations from those that did not.

Logistic regression analysis was used to describe participant characteristics associated with identifying with different symptoms under a domain that would further inquiry. Participant characteristics served as independent variables, whereas each of the 13 domains served as a dependent variable in isolation. Thus, there were 13 different models built. Assumptions for logistic regression models were met. We were unable to check for VIF and tolerance, as all the variables were collected in categorical format [22]. In the univariate analysis, all variables that were significant at ≥0.25 level of significance were included in the multivariate model. Using backward elimination criteria, only variables that had a *p*-value of <0.05 were retained in the final model [21]. Analysis was done using SAS version 9.2 [23].

## 3. Results

### 3.1. Participants Characteristics

Of the 497 participants interviewed, six did not consent to participate, leaving 491 (99%) that were included in the final analysis. The majority of participants interviewed were aged 21–25 years (35.64%), female (80.04%), current students (66.8%), of undergraduate level education (86.97%), had not been diagnosed with a mental illness before the COVID-19 pandemic (90.63%), and were not diagnosed with or treated for COVID-19 during the ongoing pandemic (84.52%) (Table 2).

### 3.2. Experience of Symptoms Mental Illness during the COVID-19 Pandemic Based on the DSM-V Criteria

A total of 449 (91.45%) participants reported symptoms of mental health disease that ranged from mild to severe and would warrant further inquiry based on the DSM-5 criteria during the COVID-19 pandemic. The proportions of participants who would warrant further investigations under each domain in isolation were as follows: depression (74.08%), mania (64.34%), anxiety (63.54%), personality functioning (62.53%), somatic symptoms (57.35%), anger (53.77%), sleep problems (49.08%), substance abuse (48.27%), repetitive thoughts and behaviors (46.64%), psychosis (43.27%), dissociation (41.55%), memory (37.88%), and suicidal ideation (6.52%) (Table 3).

Of the 466 (97%) respondents who answered questions on whether they were diagnosed with a mental disorder during the COVID-19 pandemic, 13 (2.79%) responded in the affirmative; among them, two were diagnosed with depression, two with anxiety, one with anxiety and depression, and the diagnosis for the others (8) was not documented (data not shown).

### 3.3. Factors Associated with Experiencing Symptoms of Mental Illness during the COVID-19 Pandemic That Would Warrant Further Inquiry

#### 3.3.1. Depression

A total of 74.08% of participants had symptoms of depression that warranted further investigation, with the most common symptom (63.95%) being having had little interest or pleasure in doing things during the COVID-19 pandemic in the two weeks preceding the questionnaire (Table 3). Participants with symptoms of depression did not differ from those without symptoms of depression (Table 4). 

#### 3.3.2. Mania

Overall, 64.34% of participants had symptoms of mania that warranted further inquiry. Around (49.90%) of them were bothered with starting many more projects than usual or doing more riskier things than usual for two weeks preceding the questionnaire during the COVID-19 pandemic (Table 3). Participants with symptoms of mania did not differ from those without symptoms of mania (Table 4).

#### 3.3.3. Anxiety

A total of 63.54% of participants had symptoms of anxiety that warranted further investigation, with the majority (51.73%) having been bothered with feeling nervous, anxious, frightened, worried, or on edge for two weeks preceding the questionnaire during the COVID-19 pandemic (Table 3). The odds of reporting anxiety symptoms among those aged 18–20 years were double the odds of those aged 25–30 years (OR 2.3, 95% CI 1.1–4.5, *p* = 0.02), and odds among females were almost double that of male participants (OR 1.8, 95% CI 1.1–2.8, *p* = 0.02), and the odds among those with prior mental disorders were five times more that of persons with no prior diagnosis of a mental disorder (OR 5.1, 95% CI 1.9–13.3, *p* < 0.01) (Table 4).

#### 3.3.4. Personality Dysfunction

A total of 62.53% of participants had symptoms of personality dysfunction that warranted further inquiry, with the majority (52.14%) having been bothered with not feeling close to other people or enjoying their relationships with other people for two weeks preceding the questionnaire during the COVID-19 pandemic (Table 3). Univariate analysis showed that participants with personality dysfunction were more likely to have had a prior mental disorder diagnosis than those who had not had a previous diagnosis of mental disorder (OR 5.5 95% CI 2.1–14.2, *p* < 0.01). Multivariate logistic regression was not carried out since only one factor met the criteria for inclusion in the multivariate logistic regression model (*p* < 0.25) (Table 4).

#### 3.3.5. Somatic Symptoms

Overall, 57.35% of participants had somatic symptoms that warranted further inquiry, with the majority (51.73%) having been bothered with unexplained aches and pains (e.g., head, back, joints, abdomen, legs) for two weeks preceding the survey during the COVID-19 pandemic (Table 3). The odds of reporting somatic symptoms that warranted further investigations among current students was 1.6 times that among non-students (OR 1.6, 95% CI 1.1–2.4, *p* = 0.01), and among persons with a prior diagnosis of a mental disorder, odds were 3.9 times that among persons with no prior diagnosis of a mental disorder preceding the pandemic (OR 3.7, 95% CI 1.7–8.1, *p* < 0.01) (Table 4).

#### 3.3.6. Anger

In total, 53.77% of participants had symptoms of anger that warranted further inquiry, with the majority (53.77%) having been bothered with feeling more irritated, grouchy, or angry than usual for two weeks preceding the questionnaire during the COVID-19 pandemic (Table 3). The odds of reporting anger that warranted further investigations among persons with a prior diagnosis of a mental disorder was 3.9 times that among persons with no prior diagnosis of a mental disorder preceding the pandemic (OR 3.9, 95% CI 1.8–8.3, *p* < 0.01) (Table 4).

#### 3.3.7. Sleep Problems

A total of 48.88% of participants had sleep problems that warranted further inquiry. The majority (48.88%) had been bothered with sleep quality that affected their sleep quality overall for two weeks preceding the interview during the COVID-19 pandemic (Table 3). The odds of reporting sleep problems that warranted further investigations among persons with a prior diagnosis of a mental disorder was 4.8 times that among persons with no prior diagnosis of a mental disorder preceding the pandemic (OR 4.8, 95% CI 2.3–10.3, *p* < 0.01) (Table 4).

#### 3.3.8. Substance Abuse

Overall, 48.27% of participants had symptoms of substance abuse that warranted further inquiry, with the majority (38.29%) having been bothered with using one of the following medicines on their own, that is, without a doctor’s prescription and in greater amounts or longer than prescribed (e.g., painkillers (e.g., panadol), stimulants (e.g., Adderall, codeine), sedatives or tranquilizers (e.g., sleeping pills or Valium), or drugs such as marijuana or cocaine, hallucinogens (e.g., LSD), heroin, inhalants, or solvents) for two weeks preceding the interview during the COVID-19 pandemic (Table 3). The odds of reporting substance abuse that warranted further investigations among persons with a prior diagnosis of a mental disorder was 3.4 times that among persons with no prior diagnosis of a mental disorder preceding the pandemic (OR 3.4, 95% CI 1.7–6.7, *p* < 0.01) (Table 4).

#### 3.3.9. Repetitive Thoughts and Behaviors

Overall, 46.64% of participants had repetitive thoughts and behaviors that warranted further inquiry, with the majority (40.33%) having been bothered with unpleasant thoughts, urges, or images that repeatedly entered their minds for two weeks preceding the interview during the COVID-19 pandemic (Table 3). The odds of reporting repetitive thoughts and behaviors that warranted further investigations among persons with a prior diagnosis of a mental disorder was 7.4 times that among persons with no prior diagnosis of a mental disorder preceding the pandemic (OR 7.4, 95% CI 3.2–13.9, *p* < 0.01) (Table 4).

#### 3.3.10. Psychosis

In total, 43.27% of participants had symptoms of psychosis that warranted further inquiry. The majority (37.27%) had been bothered with feeling that someone could hear their thoughts or that they could hear what another person was thinking for two weeks preceding the interview during the COVID-19 pandemic (Table 3). The odds of reporting psychosis that warranted further investigations among persons with a prior diagnosis of a mental disorder was double that among persons with no prior diagnosis of a mental disorder preceding the pandemic (OR 2.0, 95% CI 1.6–5.8, *p* < 0.01), and among those who had been diagnosed with or treated for COVID-19, the odds were almost double that of person who had not been diagnosed or treated for COVID-19 (OR 1.8, 95% CI 1.1–3.0, *p* = 0.01) (Table 4).

#### 3.3.11. Dissociation

A total of 41.55% of participants had symptoms of dissociation that warranted further inquiry, with the majority (41.55%) having been bothered with feeling detached or distant from themselves, their body, their physical surroundings, or their memories for two weeks during the COVID-19 pandemic (Table 3). The odds or reporting dissociative symptoms among females was almost double that among males (OR 1.7, 95% CI 1.0–2.8, *p* = 0.03); among persons with a prior diagnosis of a mental disorder, it was 5 times that among persons with no a prior diagnosis of a mental disorder (OR 5.1, 95% CI 2.5–10.4, *p* < 0.01); and among those who had been diagnosed with or treated for COVID-19, it was almost double that of a person who had not been diagnosed or treated for COVID-19(OR 1.7, 95% CI 1.0–2.9, *p* = 0.03) (Table 4).

#### 3.3.12. Memory Problems

A total of 37.88% of participants had symptoms of dissociation that warranted further inquiry, with the majority (37.88%) having been bothered with problems with memory (e.g., learning new information) or with location (e.g., finding their way home) for two weeks preceding the interview during the COVID-19 pandemic (Table 3). The odds of reporting memory problems that warranted further investigations among persons with a prior diagnosis of a mental disorder was 2.5 times that among persons with no prior diagnosis of a mental disorder (OR 2.5, 95% CI 1.3–4.6, *p* < 0.01) (Table 4).

#### 3.3.13. Suicidal Ideation

Overall, 6.52% (32) of participants had symptoms of dissociation that warranted further inquiry. Among the 32 participants who reported having thought of killing themselves in the week preceding the questionnaire, 75% (24) had felt that they or their families would be better off if they were dead. Almost half (46.88%; *n* = 15) had tried to kill themselves, and 46.88% (15) still had thoughts of killing themselves at the time of the interview (data not shown). Logistic regression analyses were not done due to the limited number of participants with this outcome.

## 4. Discussion

This study determined the psychological impacts of the COVID-19 pandemic amongst emerging adults (18–30), including students in Saudi Arabia, using the DSM-5 and ASQ screening tools [18]. A high proportion (91.45%) of respondents reported mental symptoms that would warrant further inquiry, and 3.26% of respondents self-reported a diagnosis of mental illness. Participants who were more likely to experience an increase in mental health symptoms were young, female students, those who had a history of being diagnosed with a mental disorder, and those diagnosed or treated for COVID-19. The majority of respondents reported depression, mania, anxiety, personality dysfunction, anger, and somatic symptoms. A minority reported substance abuse, psychosis, and suicidal ideation symptoms.

The results of this study only illustrate the proportion of participants that would require further screening either using the DSM-5 II tool or an in-depth inquiry by a mental health professional and not the actual increase in the incidence or prevalence of mental disorders during the pandemic. However, 91.45% of respondents reporting a symptom of mental health illness during the ongoing pandemic may reflect an actual increase in the incidence of mental disorders. During the COVID-19 pandemic, there has been an increase in the prevalence of mental health disorders. About 40% of adults in the U.S. reported symptoms of depression or anxiety; this rate measured over one month in 2021 quadrupled the same rate measured over six months in 2019, the pre-pandemic period [24]. In the Czech Republic, the proportion of individuals reporting a symptom of a mood disorder or substance abuse doubled during the COVID-19 pandemic [25]. The cross-sectional design employed in this study did not allow for a before-and-after comparison of respondents reporting symptoms of mental health disorders. Nevertheless, despite a high proportion of persons reporting symptoms, only a limited number may ultimately be diagnosed with a mental health disorder since this study only used preliminary screening tools [18]. The self-reported prevalence of mental disorders based on a diagnosis from a mental health professional in this study was only 3.26%. The majority of mental health disorders go undiagnosed and extend to adulthood, resulting in limited opportunities for persons with mental illness due to impaired physical and mental health [26]. Timely screening and intervention especially during uncertain times like the COVID-19 pandemic are useful to reduce the burden of mental health disease.

Emerging adults were more likely to report symptoms of mental health disease during the ongoing pandemic. The prevalence of anxiety and depressive disorders in the USA by age during the pandemic were 56%, 49%, 39%, and 29% among persons aged 18–24, 25–49, 50–64, and 65+ years, respectively [24]. Similarly, in a survey of youth aged 13 to 29 years, 27% and 15% reported anxiety and depressive behaviors in Latin America and the Caribbean [27]. In the Czech Republic, symptoms of a mood disorder or substance abuse were more common in emerging students [25]. This prevalence was partially attributed to emerging adults experiencing closures of universities and loss of jobs [24]. This increase in the prevalence of mental health disorders among emerging adults has also been seen globally [9] and in China [8]. However, the present survey did not explore why participants may have been experiencing these symptoms during the pandemic, which may help formulate interventions. Nevertheless, this study illustrated characteristics of individuals who may be the target of mental health interventions during emergencies.

Female respondents reported disproportionately higher levels of anxiety when compared to male respondents. Similar results have been observed in China [28] and Nepal [29]. In the literature, women are biologically more vulnerable to personal stresses augmented by their concern for themselves and their family members [29]. However, this study did not collect information on marital status or whether the participants had dependents.

A higher rate of psychosis symptoms was reported among persons with a prior diagnosis of a mental disorder and those diagnosed with or treated for COVID-19. New-onset psychotic symptoms have been reported in patients with COVID-19. Similarly, a high proportion of patients at fever clinics in Nepal reported symptoms of mental disorders during the COVID-19 pandemic. However, some of these patients may have already been vulnerable to psychiatric illness [29,30]. Similarly, a study in Saudi Arabia found that undergraduate students with a history of mental health disorders were prone to anxiety, depression, stress, and low levels of resilience [6]. The development of mental health disorders symptoms during the COVID-19 pandemic may have been an extension of juvenile-onset mental disorders [31]. This association is supported by the predominantly emerging adult population in this study. Nevertheless, this study did not ascertain the temporal association between a COVID-19 diagnosis or treatment and the treatment of mental health disorders.

Although close to half of the participants reported substance abuse, most consumed medications that a medical doctor had not prescribed, and only a minimal proportion had resorted to smoking or alcohol or consuming illegal drugs. An individual’s religious beliefs may influence their mental response to challenging situations. The low rates of alcohol and drug use may reflect the predominantly Islamic religion and legal prohibitions in the study area, where only 7–8% of residents report using drugs [32]. Likewise, 7% of participants had suicidal thoughts compared to 11% of adults in the U.S. [19]. In 2015, the rate of suicide for all ages in Saudi Arabia was 3.4 per 100,000 against a global average of 10.5 per 100,000 [33]. Furthermore, in the literature, suicide rates are lower among members of the Muslim religion than other religions [34]. The Islamic religion perceives suicide as sinful and damaging to a believer’s spiritual journey [35].

The strengths of this study are its large sample size and the anonymous self-administration of questionnaires, limiting the chance of social desirability bias. The study employed a detailed, updated version of the DSM-5 criteria [15]. A high overall respondent response rate and individual questions’ response rate permitted a comprehensive analysis. Nevertheless, there were some limitations. This study focused on only one unique domain at a time; however, the categories are not mutually exclusive, and participants could concurrently be placed in more than one domain. This study did not assess for all covariates that may have influenced the occurrence of mental health symptoms and participants’ access to mental health services. Furthermore, we did not follow-up with participants to assess the serial screening outcomes or diagnostic outcomes of those who were in need of further investigation. Assessing the positive predictive value of the DSM-5 Self-Rated Level 1 Cross-Cutting Symptom Measure in identifying persons who are later diagnosed with a mental illness could help its use for community mental health interventions in providing out-of-hospital care for minor symptoms of mental illness. Future research should also consider additional personal and environmental factors that may influence vulnerability to mental disorders.

## 5. Conclusions

As seen during other emergencies, this study found an increase in the prevalence of mental health symptoms among young persons during the COVID-19 pandemic. There was a high prevalence of different symptoms among specific participant groups, highlighting targeted groups for mental health interventions during epidemics and pandemics. Furthermore, it was evident that coping mechanisms may vary by an individual’s religious beliefs.

This study recommends addressing the mental health burden among young persons in time using simple, self-administered screening tools will prevent dire consequences in the future. Furthermore, emergency response plans should include funded mental health plans incorporating community health workers, universities communities, and mobile mental health services to serve as first-line mental health professionals during emergencies. Additionally, health systems should continuously relay health messages about mental health during epidemics and pandemics. Emerging adults, especially students, have experienced a significant distress during the COVID-19 lockdown that impacted their social-emotional skills and educational achievement. Therefore, emerging adults need workshops and interactive classrooms to bridge the gap of the social-emotional skills, so the universities educators need to prioritize trauma processing and pair academics to social-emotional skills in the classroom. We suggest that educators establish a safe classroom where students communicate in an empathetic and respectful way. Moreover, health systems should formulate preventive intervention plans for students and persons with a mental disorder to prevent exacerbations during periods of increased stress, such as the ongoing pandemic. Future research should also focus on the recognition and management of neuropsychiatric manifestations of mental health disorders for susceptible subjects.

## Figures and Tables

**Table 1:** DSM V Self-Rated Level 1 Cross-Cutting Symptom Measure questionnaire criteria for identifying patients that require further inquiry.

Domain	Domain Name	Threshold to Guide Further Inquiry
I.	Depression	Mild or greater
II.	Anger	Mild or greater
III.	Mania	Mild or greater
IV.	Anxiety	Mild or greater
V.	Somatic Symptoms	Mild or greater
VI.	Suicidal Ideation	Slight or greater
VII.	Psychosis	Slight or greater
VIII.	Sleep Problems	Mild or greater
IX.	Memory	Mild or greater
X.	Repetitive Thoughts and Behaviors	Mild or greater
XI.	Dissociation	Mild or greater
XII.	Personality Functioning	Mild or greater
XIII.	Substance Use	Slight or greater

Source: American Psychiatric Association.

**Table 2:** Characteristics of emerging adults (aged 18–30 years) who completed the DSM 5 and ASQ survey questions, Saudi Arabia, 2020–2021.

Participant Characteristics	Total *N* = 491 *n* (%)	Experienced Symptoms of Mental Health Disease during COVID-19 Pandemic (*n* = 449; 91.45%)	Did Not Experienced Symptoms of Mental Health Disease during COVID-19 Pandemic (*n* = 42; 8.55%)	*p*-Value
Age group				
18–20 years	148 (30.14)	137 (92.57)	11 (7.43)	0.84
21–25 years	175 (35.64)	159 (90.86)	16 (9.14)	
25–30 years	168 (34.22)	153 (91.07)	15 (8.93)	
Gender				0.87
Male	98 (19.96)	90 (91.84)	8 (8.16)	
Female	393 (80.04)	359 (91.35)	34 (8.65)	
Current student				
Yes	328 (66.80)	300 (91.46)	28 (8.54)	0.91 *
No	158 (32.18)	144 (91.14)	14 (8.86)	
Missing	5 (1.02)	5 (100.00)	0 (0.00)	
Level of education				
Undergraduate	427 (86.97)	390 (91.33)	37 (8.67)	0.82
Post-graduate	64 (13.03)	59 (92.19)	5 (7.81)	
Prior diagnosis of mental disorder				
Yes	46 (9.37)	46 (100.00)	0 (0.00)	- **
No	445 (90.63)	403 (90.56)	42 (9.44)	
Diagnosed with COVID-19				
Yes	76 (15.48)	69 (90.79)	7 (9.21)	0.82
No	415 (84.52)	380 (91.57)	35 (8.43)	

* Missing category not included in computing *p*-values. ** *p*-value not computed due to zero value in one cell.

**Table 3:** Number of participating emerging adults (aged 18–30 years) who would warrant further inquiry based on DSM-V domain criteria, Saudi Arabia, 2020–2021.

Domain	Domain Name	Number of Participants with a Threshold to Guide Further Inquiry (%)	Number of Participants with Mild or No Symptoms (%)
I.	Depression *	363 (74.08)	127 (25.92)
III.	Mania ***	314 (64.34)	174 (35.66)
IV.	Anxiety	312 (63.54)	179 (36.46)
XII.	Personality Functioning	307 (62.53)	184 (37.47)
V.	Somatic Symptoms *	281 (57.35)	209 (42.65)
II.	Anger	264 (53.77)	227 (46.23)
VIII.	Sleep Problems **	240 (49.08)	249 (50.92)
XIII.	Substance Use	237 (48.27)	254 (51.73)
X.	Repetitive Thoughts and Behaviors	229 (46.64)	262 (53.36)
VII.	Psychosis *	212 (43.27)	278 (56.73)
XI.	Dissociation	204 (41.55)	287 (58.45)
IX.	Memory	186 (37.88)	305 (62.12)
VI.	Suicidal Ideation	32 (6.52)	459 (93.48)

* missing one response, ** missing two responses, *** missing three responses.

**Table 4:** Multivariate logistic regression showing significant factors associated with reporting symptoms of mental disorders that warrant further inquiry, Saudi Arabia, 2021.

**Participant Characteristics**	**Anxiety Domain** **OR (95% CI)**	**Somatic Symptoms** **OR (95% CI)**	**Anger** **OR (95% CI)**	**Sleep Problems** **OR (95% CI)**	**Substance Use** **OR (95% CI)**	**Repetitive Thoughts and** **Behaviors** **OR (95% CI)**	**Psychosis** **OR (95% CI)**	**Dissociation** **OR (95% CI)**	**Memory** **OR (95% CI)**
Age group: 18–20 years vs. 25–30 years	2.3 (1.1–4.5)								
Female vs. male gender	1.8 (1.1–2.8)								
Current student vs. non-student status		1.6 (1.1–2.4)		1.5 (1.0–2.1)					
Education level									
Prior diagnosis of mental disorder	5.1 (1.9–13.3)	3.7 (1.7–8.1)	3.9 (1.8–8.3)	4.8 (2.3–10.3)	3.4 (1.7–6.7)	7.4 (3.2–16.9)	2.0 (1.6–5.8)	5.1 (2.5–10.4)	2.5 (1.3–4.6)
Diagnosed with or treated for COVID-19							1.8 (1.1–3.0)	1.7 (1.0–2.9)	

Logistic regression analysis was used to describe participant characteristics associated with identifying with different symptoms under a domain that would further inquiry. Participant characteristics served as independent variables, whereas each of the 13 domains served as a dependent variable in isolation. Thus, there were 13 different models built. A limited number of participants prevented further stratified analyses to assess for interactions. No participant factors were retained in the final models for depression, mania, personality functioning, and suicidal ideation.

## Data Availability

The datasets used and/or analyzed during the current study are available from the corresponding author on reasonable request.

## Supplementary Materials

The following supporting information can be downloaded at: https://www.mdpi.com/article/10.3390/ijerph19031445/s1, Table S1: Proportions of participants with symptoms of mental health disease during the pandemic.
