# Supplementary material for: The Psychological Impacts of COVID-19 Pandemic among Emerging Adults: An Observational Cross-Sectional Study
Source: Int J Environ Res Public Health. 2022 Jan 27;19(3):1445. doi: 10.3390/ijerph19031445 (PMC8835027; doi:10.3390/ijerph19031445)
Supplement: Supplementary file 1 [file ijerph-19-01445-s001.zip › ijerph-1518165-supplementary.pdf]

Supplementary Table 1. Proportions of participants with symptoms of mental health disease during the pandemic.

Symptom	Participant Experience ¥	
	Warrants further inquiry (%)	Doesn't warrant further inquiry (%)
Depression		
How many days in the last 2 weeks have you had little interest or pleasure in doing things during the COVID-19 pandemic?	314 (63.95)	177 (36.05)
How much have you been bothered with feeling down, depressed, or hopeless for a period of TWO WEEKS during the COVID-19 pandemic? *	292 (59.47)	198 (40.33)
Mania		
How much have you been bothered with sleeping less than usual, but still have a lot of energy for a period of TWO WEEKS during the COVID-19 pandemic? ***	235 (47.86)	253 (51.53)
How much have you been bothered with starting lots more projects than usual or doing more risky things than usual for a period of TWO WEEKS during the COVID-19 pandemic?	245 (49.90)	246 (50.10)
Anxiety		
How much have you been bothered with feeling nervous, anxious, frightened, worried, or on edge for a period of TWO WEEKS during the COVID-19 pandemic?	254 (51.73)	237 (48.27)
How much have you been bothered with avoiding situations that make you anxious for a period of TWO WEEKS during the COVID-19 pandemic?	243 (49.49)	248 (50.51)
How much have you been bothered with feeling panic or being frightened for a period of TWO WEEKS during the COVID-19 pandemic?	231 (47.05)	260 (52.95)
Personality functioning		
How much have you been bothered with not feeling close to other people or enjoying your relationships with them for a period of TWO WEEKS during the COVID-19 pandemic?	256 (52.14)	235 (47.86)
How much have you been bothered with not knowing who you really are or what you want out of life for a period of TWO WEEKS during the COVID-19 pandemic?		
Somatic symptoms		
How much have you been bothered with Unexplained aches and pains (e.g., head, back, joints, abdomen, legs) for a period of TWO WEEKS during the COVID-19 pandemic?	254 (51.73)	237 (48.27)

How much have you been bothered with feeling that your illnesses are not being taken seriously enough for a period of TWO WEEKS during the COVID-19 pandemic? *	159 (32.38)	331 (67.41)
Anger		
How much have you been bothered with feeling more irritated, grouchy, or angry than usual for a period of TWO WEEKS during the COVID-19 pandemic?	264 (53.77)	227 (46.23)
Sleep problems		
How much have you been bothered with problems with sleep that affected your sleep quality over all for a period of TWO WEEKS during the COVID-19 pandemic? **	240 (48.88)	249 (50.71)
Substance abuse		
How much have you been bothered with using any of the following medicines ON YOUR OWN, that is, without a doctor's prescription, in greater amounts or longer than prescribed [e.g., painkillers (like panadol), stimulants (like Adderall, codeine), sedatives or tranquilizers (like sleeping pills or Valium), or drugs like marijuana, or cocaine, hallucinogens (like LSD), heroin, inhalants or solvents] for a period of TWO WEEKS during the COVID-19 pandemic?	188 (38.29)	303 (61.71)
How much have you been bothered with smoking any cigarettes, a cigar, or pipe, or using snuff or chewing tobacco for a period of TWO WEEKS during the COVID-19 pandemic?	141 (28.72)	350 (71.28)
How much have you been bothered with drinking at least 4 drinks of any kind of alcohol in a single day for a period of TWO WEEKS during the COVID-19 pandemic?	94 (19.14)	397 (80.85)
Repetitive thoughts and behaviors		
How much have you been bothered with Unpleasant thoughts, urges, or images that repeatedly enter your mind for a period of TWO WEEKS during the COVID-19 pandemic?	198 (40.33)	293 (59.67)
How much have you been bothered with Feeling driven to perform certain behaviours or mental acts over and over again for a period of TWO WEEKS during the COVID-19 pandemic?	169 (34.42)	322 (65.58)
Psychosis		
How much have you been bothered with feeling that someone could hear your thoughts, or that you could hear what another person was thinking for a period of TWO WEEKS during the COVID-19 pandemic?	183 (37.27)	308 (62.73)
How much have you been bothered with hearing things other people couldn't hear, such as voices even when no one was around for a period of TWO WEEKS during the COVID-19 pandemic? *	172 (35.03)	318 (64.77)
Dissociation		

How much have you been bothered with feeling detached or distant from yourself, your body, your physical surroundings, or your memories for a period of TWO WEEKS during the COVID-19 pandemic?	204 (41.55)	287 (58.45)
Memory		
How much have you been bothered with problems with memory (e.g., learning new information) or with location (e.g., finding your way home) for a period of TWO WEEKS during the COVID-19 pandemic? ****	186 (37.88)	305 (62.12)
Suicidal ideation	Yes	No
In the past week, have you been having thoughts about killing yourself?	32 (6.52)	459 (93.48)

*Missing 1 response **Missing 2 responses , **** missing 3 responses; ¥ Pyschosis, Suidical ideation and substance abuse: 1) Slight- Rare, less than a day or two ; 2) Mild- Several days; 3) Moderate- More than half the days; 4) Severe- Nearly every day all warrant further inquiry. 0) None- Not at all does not warrant further inquiry. All other domains: 2) Mild- Several days; 3) Moderate- More than half the days; 4) Severe- Nearly every day all warrant further inquiry. 0) None- Not at all & 1) Slight- Rare, less than a day or two do not warrant further inquiry.
